# On manufacturing multilayer-like nanostructures using misorientation gradients in PVD films

**DOI:** 10.1038/s41598-019-52226-1

**Published:** 2019-11-04

**Authors:** Pedro Renato Tavares Avila, Erenilton Pereira da Silva, Alisson Mendes Rodrigues, Katherine Aristizabal, Fabiola Pineda, Rodrigo Santiago Coelho, Jose Luís Garcia, Flavio Soldera, Magdalena Walczak, Haroldo Cavalcanti Pinto

**Affiliations:** 1 Department of Materials Engineering - SMM, São Carlos School of Engineering – EESC, University of São Paulo – USP, São Carlos, SP Brazil; 2 Institute of Engineering, Science and Technology (IECT), Federal University of Vales do Jequitinhonha e Mucuri (UFVJM), Janaúba-MG, 39447-790 Brazil; 3 Institute of Functional Materials, Department of Materials Science and Engineering, Saarland University, Saarbrücken, Germany; 4 Department of Mechanical and Metallurgical Engineering, Escuela de Ingeniería, Pontificia Universidad Católica de Chile, Vicuña Mackenna, 4860 Santiago, Chile; 5 Institute of Innovation for Forming and Joining of Materials, SENAI CIMATEC, Salvador, BA 41650-010 Brazil; 6 Sandvik Machining Solutions, R&D Material & Processes, Lerkrogsvägen 19, SE 12160 Stockholm, Sweden

**Keywords:** Surfaces, interfaces and thin films, Synthesis and processing

## Abstract

Due to their applicability for manufacturing dense, hard and stable coatings, Physical Vapor Deposition (PVD) techniques, such as High Power Impulse Magnetron Sputtering (HiPIMS), are currently used to deposit transition metal nitrides for tribological applications. Cr-Al-N is one of the most promising ceramic coating systems owing to its remarkable mechanical and tribological properties along with excellent corrosion resistance and high-temperature stability. This work explores the possibility of further improving Cr-Al-N coatings by modulation of its microstructure. Multilayer-like Cr_1−x_Al_x_N single films were manufactured using the angular oscillation of the substrate surface during HiPIMS. The sputtering process was accomplished using pulse frequencies ranging from 200 to 500 Hz and the resulting films were evaluated with respect to their hardness, Young’s modulus, residual stresses, deposition rate, crystallite size, crystallographic texture, coating morphology, chemical composition, and surface roughness. The multilayer-like structure, with periodicities ranging from 250 to 550 nm, were found associated with misorientation gradients and small-angle grain boundaries along the columnar grains, rather than mesoscopic chemical modulation of the microstructure. This minute modification of microstructure along with associated compressive residual stresses are concluded to explain the increased hardness ranging from 25 to 30 GPa, which is at least 20% over that expected for a film of the same chemical composition grown by a conventional PVD processing route.

## Introduction

Surfaces of engineering workpieces are often modified by thermal and thermochemical means to improve the tribological and corrosion properties. Surface treatments involve the application of specific thermochemical cycles to a material to obtain desired mechanical and chemical properties^1,2^. In addition, the deposition of films with specific properties has proved advantageous as a further step towards optimum engineering surfaces^2^.

The most employed methods of thin film deposition for the modification of engineering surface properties are chemical vapor deposition (CVD), chemical vapor deposition assisted by plasma (PE-CVD) and physical vapor deposition (PVD). The latter category is very broad, and the High Power Impulse Magnetron Sputtering (HiPIMS) technology represents one of the most recent advances in terms of physical film deposition.

Ceramic coatings are used to protect manufactured parts from thermal and/or corrosive degradation, confer wear resistance by enhancing surface hardness and may diminish friction-associated losses while maintaining toughness and ductility of the core material. Hence, typical applications of hard ceramic coatings are inner surfaces of combustion engines, working surfaces of cutting tools and forming dies, among others^3^. Transition Metal (TM) nitride films are valuable materials owing to their attractive set of properties, such as thermal conductivity, wear, and chemical resistance, as well as their appearance and esthetic appeal to the customers^4–6^. Among the TM nitrides, CrN exhibits great potential as a film for tribological applications due to its remarkable hardness, wear and corrosion resistance as well as refractory properties, e.g^7^. Rock salt fcc-structured CrN coatings are used in automotive combustion engines, e.g.^8,9^. The addition of Al to the CrN lattice, building up a substitutional solid solution with Cr and having the general stoichiometry of Cr_1−x_Al_x_N, can improve the mechanical properties, thermal stability, and wear behavior of CrN-based films^10–12^.

Coatings can be engineered to produce superior properties by tailoring deposition techniques and parameters. The development of multilayered films has been shown effective for improving the mechanical properties, such as hardness, toughness and elastic modulus. Several multilayer architectures with compositional alternation between sub-layers were reported to improve hardness and physical properties of electrical conductivity and difussional barrier^13–15^. The improvements in mechanical properties of hardness, toughness and stiffness, were found associated with the formation of superlattice structures and with a higher density of layer boundaries that obstruct plastic deformation throughout the coating by acting as a barrier for dislocation gliding^16^.

Builiding on the above premises, this work proposes an alternative approach for modyfing mechanical properties through modulation of misoritentation perpendicular to the engnineered surface. The misorientation gradients are introduced into the Cr_1−x_Al_x_N film architecture by implementing the angular oscillation of the substrate surfaces in front of the Cr_50_Al_50_ (at%) targets during HiPIMS. The impact of HiPIMS pulse frequencies ranging from 200 to 500 Hz is evaluated for the same Cr_1−x_Al_x_N film stoichiometry with a crystalline phase indexed as fcc-Cr_1−x_Al_x_N.

We believe that the excellent mechanical performance of our multilayer-like nanostructured fcc-Cr_1−x_Al_x_N single films along with the feasibility and scalability towards industrial, will contribute to develop optimized technologies for the upcoming demands of protective layers for high-performance combustion engines with respect to lowest pollutant emissions.

## Results and Discussion

### Coatings morphology

**Figure 1:**
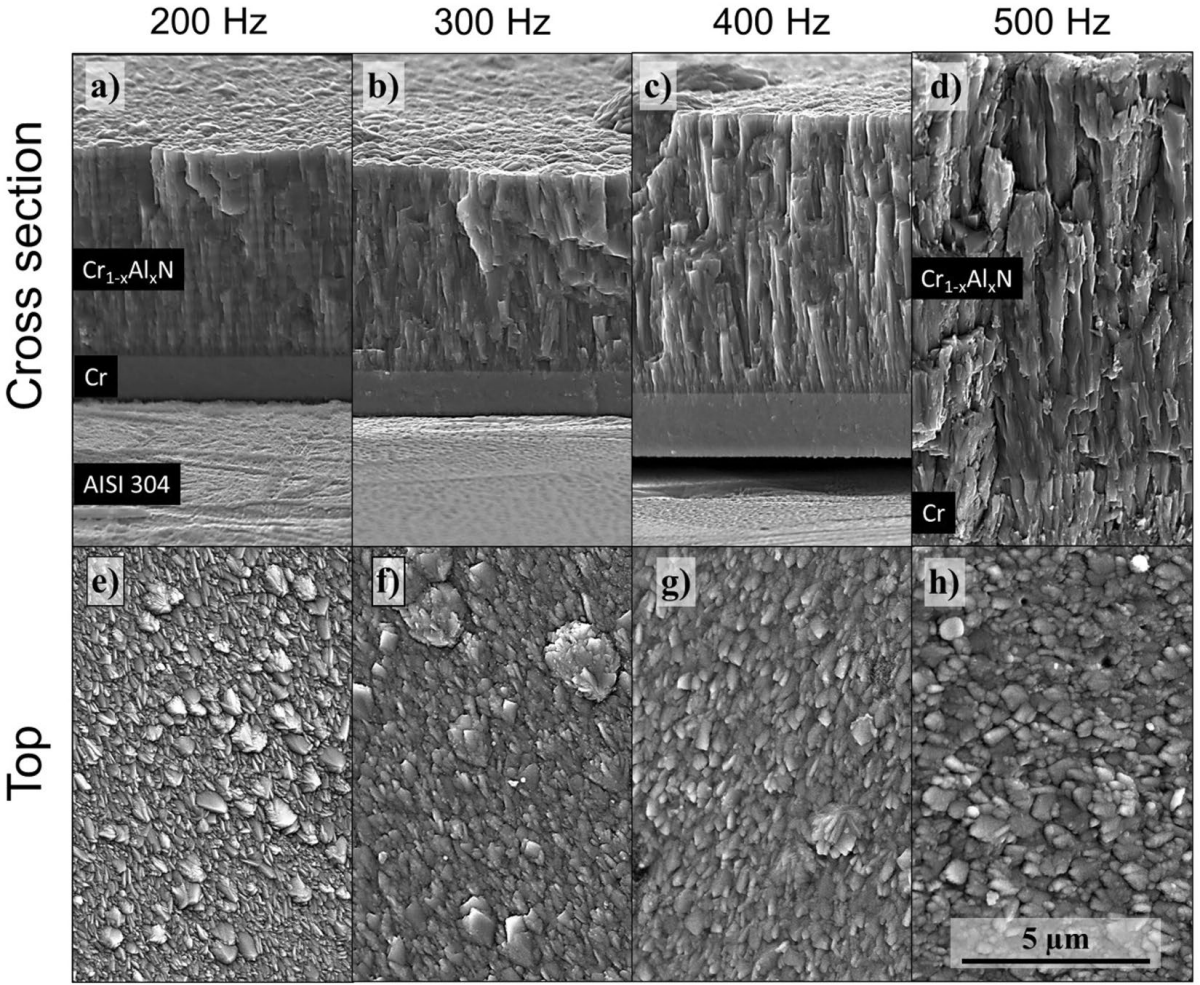
Morphology of the cross-sections and top surface observed in FEG-SEM for Cr_1−x_Al_x_N single films deposited by HiPIMS using the indicated pulse frequencies.

SEM images were acquired from the fracture cross-section and top surface of the Cr_1−x_Al_x_N films manufactured by HiPIMS (Fig. 1). At the SEM images of coating fracture cross-section (Fig. 1a–d), it was possible to observe that the films presented a dense and compact structure with columnar grains, which is the typical morphology from zone T in a structure zone model^17^. FEG-SEM images obtained from the top surface (Fig. 1e–h) confirm the formation of dense films without pores. In addition, morphological cauliflower-like inclusions, common in sputtering processes, are present and randomly distributed on top of the Cr_1−x_Al_x_N films.

**Figure 2:**
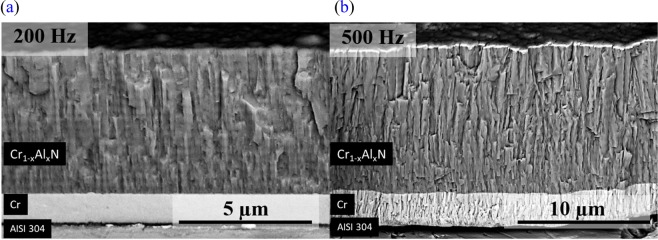
Cross-sectional FEG-SEM fracture images of Cr_1−x_Al_x_N film deposited by HiPIMS at 200 Hz (**a**) and 500 Hz (**b**). The multilayer-like architecture is present in both images.

**Figure 3:**
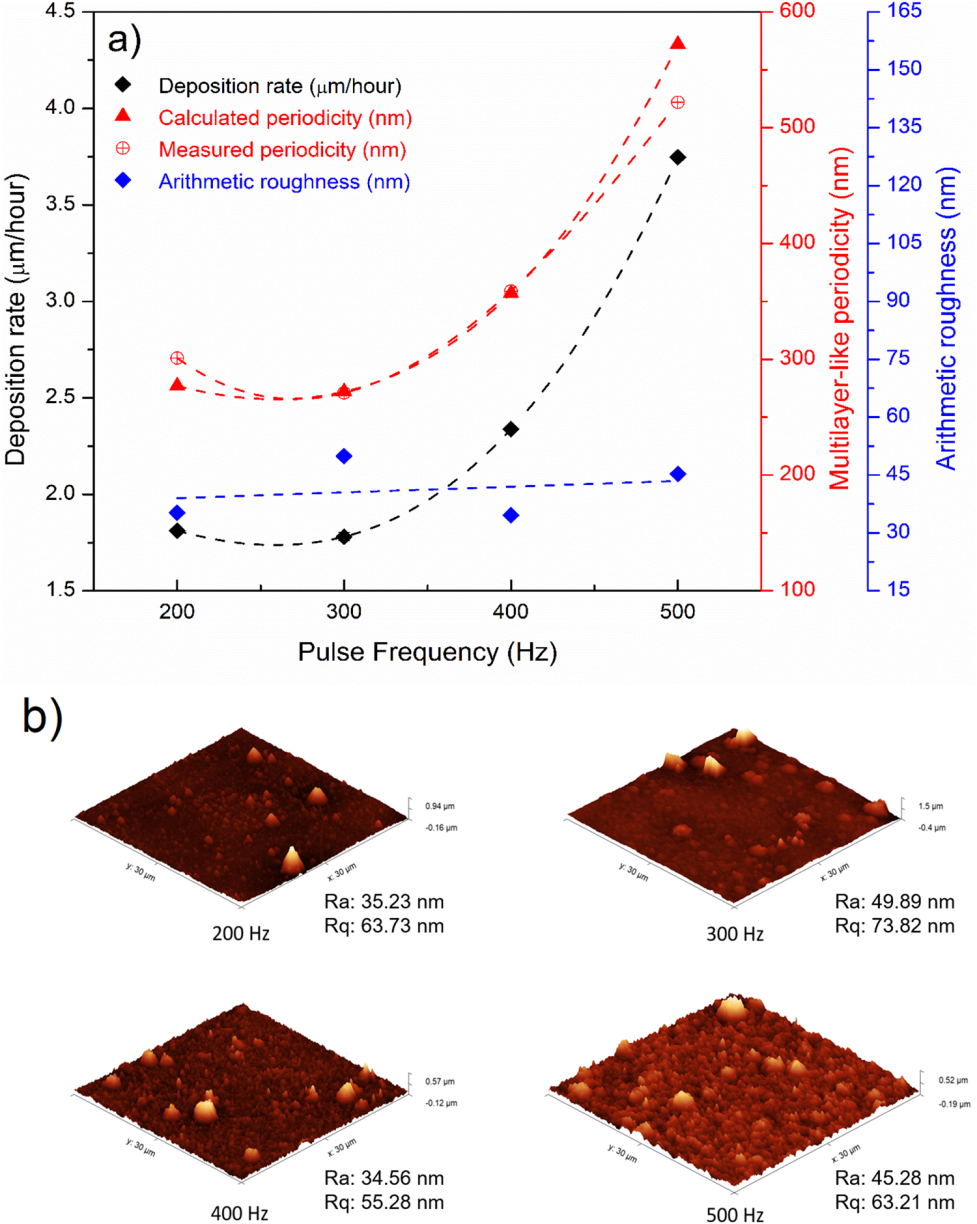
(**a**) Pulse frequency dependence of the deposition rate for Cr_1−x_Al_x_N films, (**b**) 3D AFM surface maps from the Cr_1−x_Al_x_N films deposited at 200 Hz, 300 Hz, 400 Hz and 500 Hz.

As displayed in Fig. 2, the thickness of the coatings deposited at 500 Hz roughly double that of 200 Hz indicating that the deposition rate increases when pulse frequency is incremented (Fig. 3). The dependence of deposition rate and pulse frequency is consistent with previous studies^18–20^. Bobzin *et al*.^18^ measured an increase in metal ionization for shorter frequencies due to higher power peaks. This enhances the self-sputtering mechanism, in which ionized particles are back attracted to the target instead of contributing to the film growth, thus reducing the ion flux to the substrate. Along with that, ionized particles are accelerated when substrate bias is present. Since this was the case for all Cr_1−x_Al_x_N films deposited in this work, a higher degree of ionization at lower frequencies produces a higher number of accelerated particles arriving at the substrate. Since the energy of bombardment on the growing film is greater in this case, it causes re-sputtering of material already condensed on the substrate surface, and hence reduces the deposition rate^21^.

The multilayer-like architecture was observed throughout the cross-sectional view of the Cr_1−x_Al_x_N single films in which horizontal stripes of higher and lower brightness, causing a wavy profile along the columnar grain boundaries, are apparent, most notably in Fig. 2a. The zig-zag morphology of grain growth can be related to the oscillatory motion of the steel discs in front of the Cr_50_Al_50_ (at.%) target during deposition. The multilayer-like architecture obtained by substrate oscillation bear some resemblance with helicoidal structures produced by Glancing Angle Deposition (GLAD)^22,23^, however, due to the high angles of deposition usually involved in the later, and to the unavoidable shadowing effect, GLAD coatings tend to be less dense and compact. During oscillation of the substrate surface, the low angular amplitude and constant angular motion avoid shadowing effects, and the coatings present a compact structure, similar to depositions at a normal incidence angle.

Figure 3 summarizes information regarding deposition rate, calculated and measured multilayer-like periodicities as well as mean surface roughness achieved for each pulse frequency and considering the oscillatory motion with 120 s period. The theoretical periodicity values were predicted using Eq. 1, with *t* being the multilayer-like periodicity, *r* the deposition rate (shown in Fig. 3) and *p* the period of oscillatory motion. The measured and calculated periodicities are in good agreement and support the assumption of zig-zag orientation of growth due to the oscillatory motion of the substrate surface in front of the target during deposition.

1 $$t=r\ast p$$

The measured and calculated periodicities are in good agreement and support the assumption of zig-zag orientation of growth due to the oscillatory motion of the substrate surface in front of the target during deposition. The dependence of the mean arithmetic surface roughness (Ra) on the pulse frequency was determined from 3D AFM analyses (Fig. 3b). As displayed in Fig. 3a, all Cr_1−x_Al_x_N films exhibited low Ra values. In addition, no systematic change in surface finishing can be attributed to the variation in pulse frequency.

### Chemical composition

**Figure 4:**
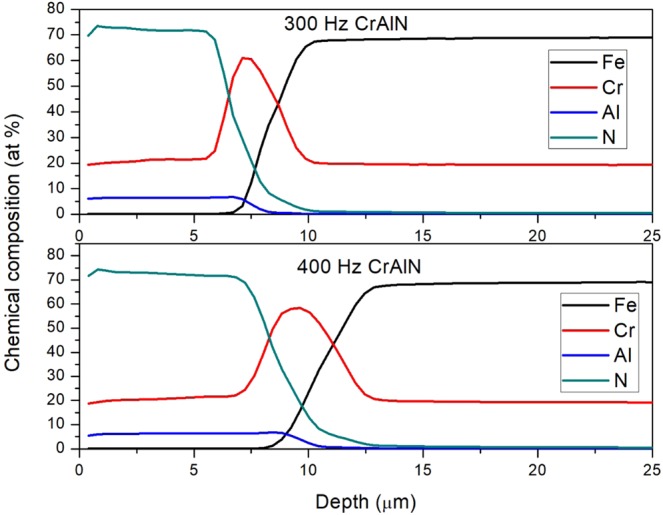
GDOES depth profile of chemical composition throughout the multilayer-like Cr_1−x_Al_x_N single film and the Cr base layer deposited onto AISI 304 L steel substrate by HiPIMS at 300 Hz and 400 Hz. Transitions from Cr_1−x_Al_x_N film to the Cr base layer and the steel substrate are evident.

The chemical composition of all Cr_1−x_Al_x_N films was determined using GDOES. All coatings presented a similar chemical composition profile, and no dependency on pulse frequency was verified. The GDOES analyses illustrated in Fig. 4 were carried out for the Cr_1−x_Al_x_N single films deposited at 300 Hz and 400 Hz. The chemical composition of the coating is not stoichiometric and the Cr/Al ratio (20/5 at.%) does not coincide with that of the target used in deposition (50/50 at.%). Moreover, there are elevated levels of nitrogen, which can be explained by the N_2_ and Ar gases used during deposition (flow rate of 50 sccm and 40 sccm, respectively). The transitions from the Cr_1−x_Al_x_N film to the Cr base layer and then to the AISI 304 L steel substrate are visible. No effects of the multilayer-like structure are present in the chemical depth profile, thus indicating that there are no relevant chemical changes throughout the multilayer-like architecture.

### Microstructure evaluation

**Figure 5:**
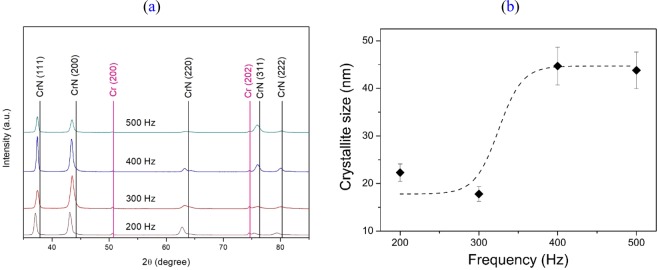
(**a**) θ-2θ x-ray diffractograms from multilayer-like Cr_1−x_Al_x_N single films deposited by HiPIMS at different pulse frequencies (200 Hz, 300 Hz, 400 Hz, and 500 Hz); (**b**) Crystallite size dependence on the HiPIMS pulse frequency for Cr_1−x_Al_x_N multilayer-like single films (200 Hz, 300 Hz, 400 Hz, and 500 Hz).

XRD phase analyses were performed using θ-2θ diffractograms to characterize the Cr_1−x_Al_x_N films deposited at different pulse frequencies (200 Hz, 300 Hz, 400 Hz, and 500 Hz), see Fig. 5.

Besides the peaks relative to the metallic Cr base layer, all peaks were identified as belonging to fcc-Cr_1−x_Al_x_N. This demonstrates that no hcp-AlN was built up and the entire Al-content was maintained in the solid solution of the fcc-Cr_1−x_Al_x_N phase. Moreover, no hcp-Cr_2_N phase was encountered due to the elevated N_2_/Ar ratio used during HiPIMS.

All peaks present a shift to lower 2θ angles, if compared to the reference fcc-CrN lines, with lower shifts occurring for higher frequencies. Besides a reduction of lattice parameter as a result of Al-addition, it indicates that in the surface normal direction (ND), i.e. the direction of film growth, the lattice spacings are increased. This is likely due to a Poisson-conditioned transversal expansion caused by the existence of elevated in-plane compressive residual stresses, which are typically generated by ion bombardment of coatings during PVD processes.

All multilayer-like Cr_1−x_Al_x_N single films exhibited strong (111)/(200) fiber texture components, both typical in the growth of CrN films^24^, as they have higher atomic packing density and, therefore, lower surface energy in B1 NaCl structure^25^. However, the strain energy also matters and can be minimized during film condensation by diffusion of arriving material to lower density planes^10^. When pulse frequency decreases, the power peaks increase to maintain the average power density, and the degree of ionization is also enhanced. As a result of this process, more energy is provided to the growing film by momentum transfer during collisions, thus enhancing the mobility of adatoms in the surface by substrate heating. Owing to the higher mobility and kinetic energy of atoms, diffusion may take place to minimize strain energy by the growth of less dense planes that can allocate sputtered atoms with lower lattice distortion. This effect can be observed in Fig. 5a, as peak intensity corresponding to (220) plane increases and to (311) decreases when the pulse frequency is reduced from 500 Hz to 200 Hz. Still, no substantial texture evolution can be observed, as it was reported before^18,19,26^.

The evolution of crystallite size with the HiPIMS pulse frequency is displayed in Fig. 5b. It can be noticed that there is an influence of frequency on crystallite size. The increase of crystallite size with frequency was also observed by^21^. This was associated with the less energetic bombardment of the growing film due to the lower ratio of ion to neutral atom during sputtering in conditions of high pulse frequency. Hence, a smaller number of grain boundaries will be formed and, thus, there will be larger crystallites. In contrast to the microstructure coarsening, the lower power peaks generated by high pulse frequencies lead to fewer lattice imperfections and lattice distortion during the crystallite growth.

### TEM evaluation of multilayer-like structures in Cr_1−x_Al_x_N films

**Figure 6:**
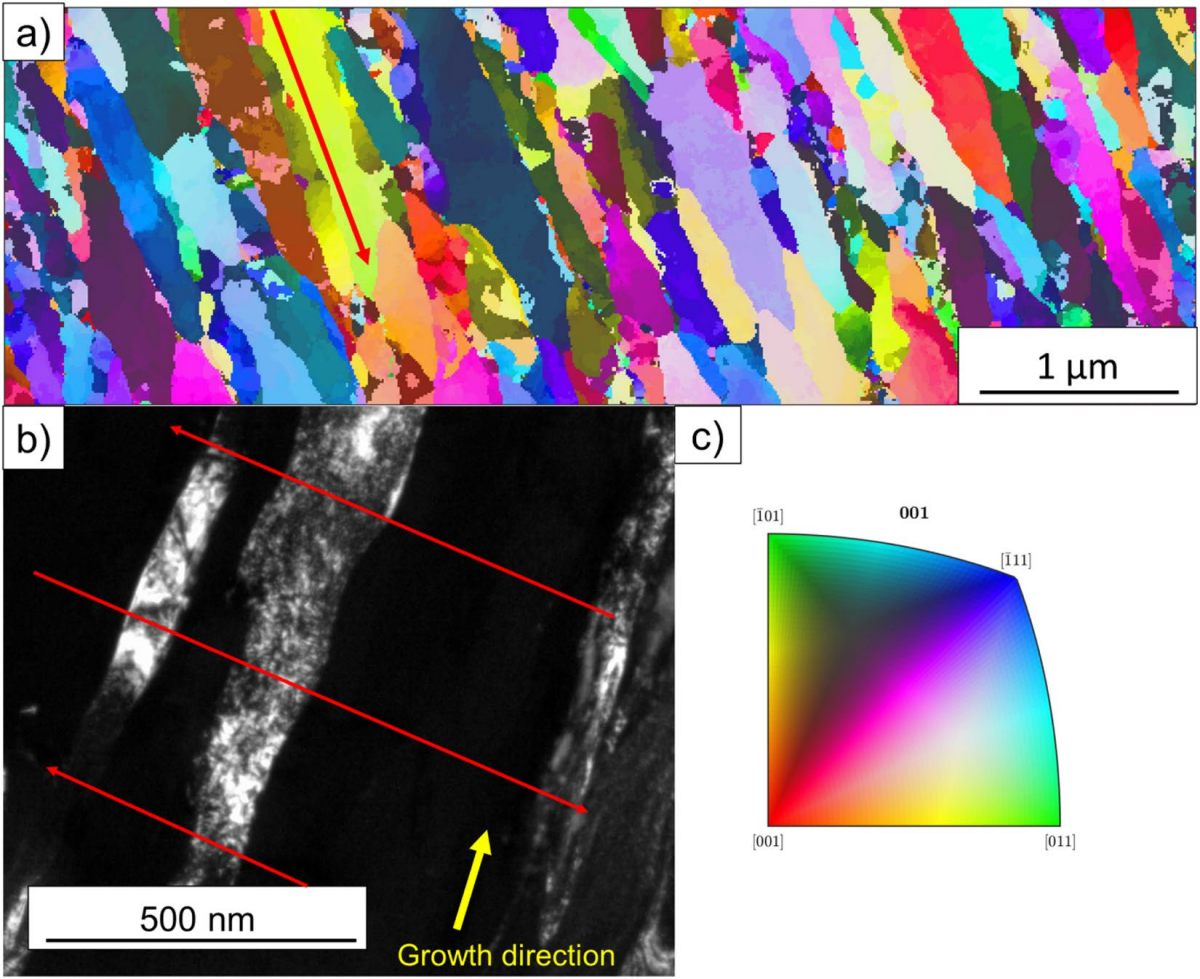
High resolution characterization of the multilayer-like Cr_1−x_Al_x_N single film deposited by HiPIMS at 200 Hz: (**a**) IPF map of multilayer-like Cr_1−x_Al_x_N single film deposited by HiPIMS obtained by precession electron diffraction; (**b**) dark field TEM image, where red arrows indicate the zig-zag morphology of grain growth responsible for the multilayer-like structure observed in previous FEG-SEM images; and (**c**) color key for the stereographic standard triangle of cubic structures.

To further understand the formation and nature of the multilayer-like architecture observed in the FEG-SEM images (Fig. 2), TEM analyses were conducted. Figure 6a reports an Inverse Pole Figure (IPF) map with respect to the direction of film growth obtained via precession electron diffraction of the TEM lamella from the multilayer-like Cr_1−x_Al_x_N single film deposited by HiPIMS at 200 Hz. Blue and purple grains corresponding to planes with orientation between (001) and (111) are in larger fraction, in accordance with the strongest fiber textures observed in the XRD analyses (Fig. 5). The presence of in-grain misorientation is visible in the IPF map as color gradients within the columnar grains.

The dark field TEM image of the Cr_1−x_Al_x_N film deposited at 200 Hz (Fig. 6b) reveals that the columnar grains exhibit a zig-zag morphology of grain growth (red arrows), which are consistent with the multilayer-like periodicities measured using FEG-SEM and calculated based on Eq. (1) and (Fig. 3). However, in Fig. 6, image analyses indicate that the period of those zig-zag structures is roughly 275 nm (distance between two red arrows). This is in good agreement with the results of calculated periodicities in Fig. 3 and confirms the correlation between the angular oscillation of substrate surfaces and the zig-zag morphology of grain growth. One can notice that there are no abrupt change or interface between the sub-layers, as in conventional multi-layers, but rather a smooth change in the direction of grain growth, which resembles the formation of small-angle grain boundaries (SAGB) within the columnar grains. This is expected due to the continuous motion of the substrate surface along 10° of oscillation amplitude. In addition, the grain boundaries, as a result of the zig-zag morphology of grain growth, evolve a corrugated arrangement, as it can be also visualized in Fig. 6b.

**Figure 7:**
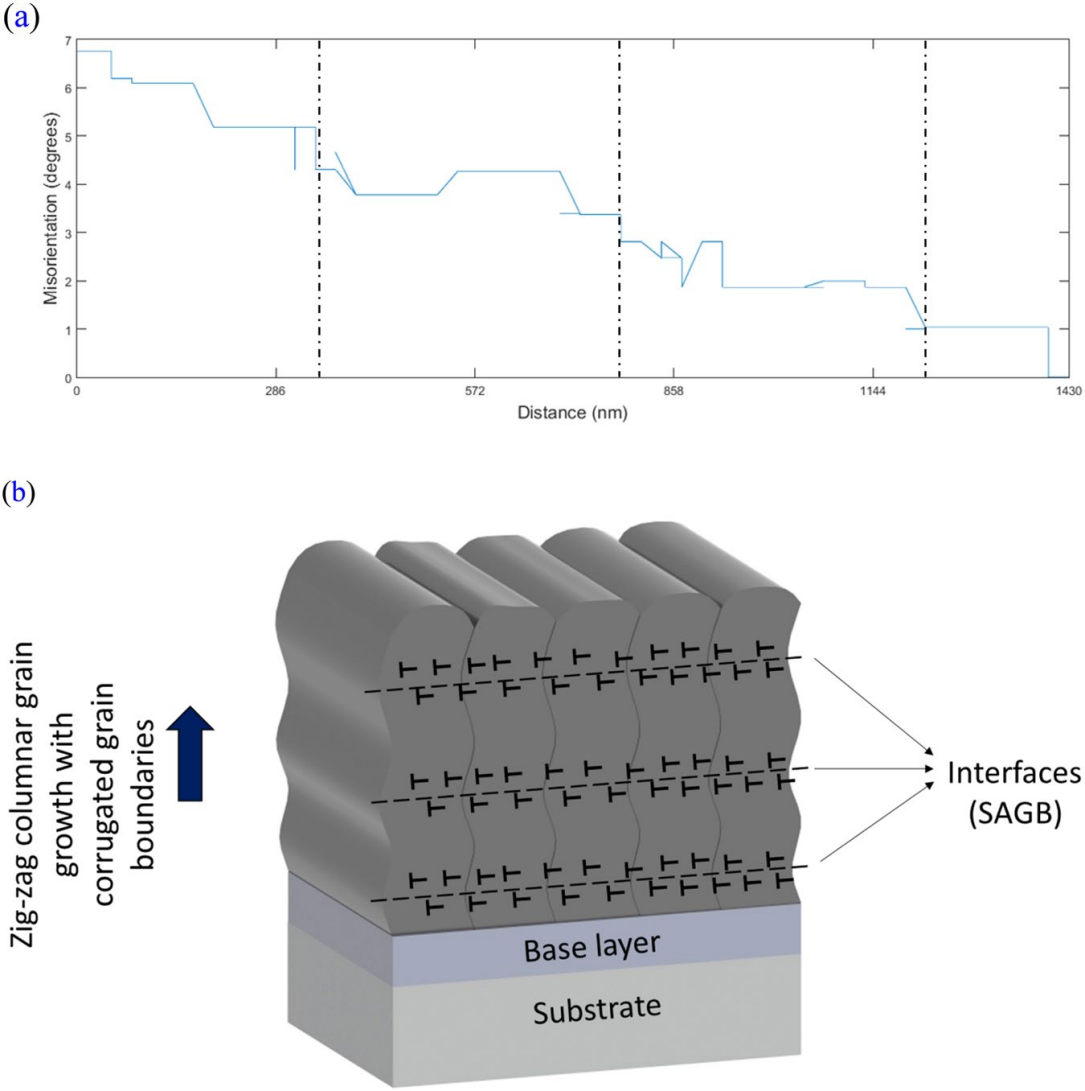
(**a**) Misorientation versus distance plot corresponding to the red arrow in Fig. 6a. It shows in detail the misorientation gradient of a few degrees and its periodic decrease related to the multilayer-like architecture. (**b**) Schematic representation of the microstructure defining a multilayer-like single film.

To quantify the misorientation gradient inside the columnar grains, a misorientation plot is displayed in Fig. 7a. These measurements were performed along a single columnar grain in the direction of film growth, as detailed by the red arrow in Fig. 6a. It can be observed that there is a gradient of misorientation along the columnar grains of a few degrees. It is also noticeable that the angular shift decreases in periodic steps. These steps are of the same order of magnitude as the multilayer-like periodicities induced by the zig-zag structures measured in Fig. 6b and coincide with the measured and calculated periodicity for 200 Hz pulse frequency, i.e. approximately 275 nm. This indicates that the multilayer-like architecture observed by FEG-SEM and TEM analyses are formed by shifts of a few degrees in the direction of grain growth, which is caused by differences in incidence angle of the sputtered ions, since the target is always stationary, and the substrate surface is oscillating with a period of −5°/+5°. This creates a misorientation gradient which generates, however, SAGB and is not enough to interrupt the columnar growth or cause the nucleation of new grains, as can be seen on the Fig. 7b.

### Mechanical properties

**Figure 8:**
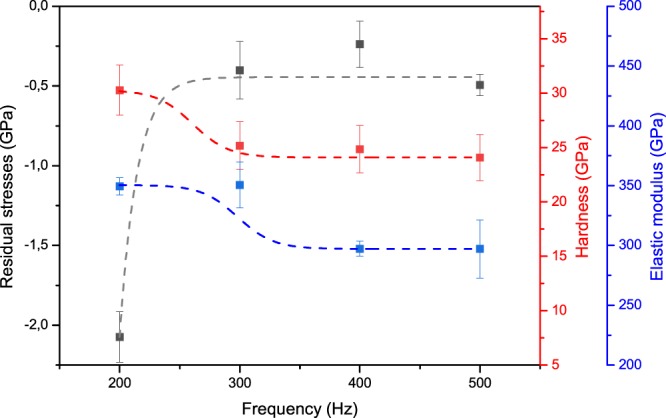
Correlation between pulse frequency used for deposition of multilayer-like Cr_1−x_Al_x_N single films and the resulting in-plane residual stresses, hardness, and elastic modulus.

Figure 8 shows the influence of pulse frequency on the mechanical properties of multilayer-like Cr_1−x_Al_x_N single films, such as residual stresses, hardness, and elastic modulus. All Cr_1−x_Al_x_N films exhibited compressive residual stresses. The compressive stresses diminish as pulse frequency rises from 200 to 300 Hz. This behavior has been reported by other authors^18^ and can also be explained by the change in ionization degree with the HiPIMS pulse frequency. As the bombardment energy decreases at higher frequencies, the in-plane film strain will decrease owing to the reduction of lattice displacements, gas implantation within the film, induction of substitutional or interstitial defects and other growth defects that contribute to intrinsic residual stresses in PVD coatings. This corroborates the lower compressive stress levels for the films deposited at higher frequencies^24^.

These results are also in good agreement with the diffraction line shifts to lower 2θ angles and the lower 2θ shifts observed for higher frequencies, which are clearly visible in the θ-2θ diffractograms measured in the surface ND, i.e. the direction of film growth (Fig. 5a). This is associated with Poisson-related expansion in the surface ND as a result of compressive in-plane residual stresses.

Since Cr_1−x_Al_x_N films deposited at lower frequencies presented higher compressive stress levels and higher mobility of atoms due to more elevated power peaks applied to the sputtering targets, diffusion may take place to minimize strain energy by the growth of less dense lattice planes that can allocate dislodged atoms with lower lattice distortion. This can explain the occurrence of (220) and (311) fiber texture components in Fig. 5a, as the HiPIMS pulse frequency is diminished from 500 to 200 Hz.

Although previous works^18^ reported a trend of increasing coating hardness with increasing pulse frequency or no influence of frequency on hardness, even though compressive residual stresses decrease^19^, this was not the case in this study as hardness along with the compressive residual stresses exhibited a trend to decrease for increasing frequencies (Fig. 8). One of the reasons suggested to explain this behavior is the lack of expressive texture changes for different frequencies, as observed previously^21^. Moreover, there can be a significant effect of the multilayer-like structures described previously. Since they are formed by misorientation gradients, and consequently by SAGB inside the columnar grains, the multilayer-like architecture contributes to the formation of additional intrinsic residual stresses and interferes with dislocation gliding and plastic deformation during indentation. Hence, as the sub-layers become thicker for higher frequencies, i.e. higher deposition rates (Fig. 3), the effect of misorientation gradients may become less important, and their impact on hardness and compressive stresses are less evident.

## Conclusions

Dense and compact Cr_1−x_Al_x_N films were obtained with multilayer-like structure defined by minute microstructure modulation perpendicular to the surface. The films are characterized by hardness at least 20% higher over conventional film structure, which was shown tunable between 25 and 30 GPa for the periodicities ranging from 250 to 550 nm, respectively. This enhancement in mechanical properties is associated with elevated compressive residual stresses and an increased number of obstacles to dislocation gliding as shown for the low HiPIMS pulse frequencies. The reduction in pulse frequency produces more energetic film deposition, thus diminishing deposition rate and crystallite size. The latter contributes to increase coating hardness. No influence of pulse frequency was observed on the surface finishing of the films. The multilayer-like architecture was manufactured by angular oscillation of the substrate surface in front of the sputtering target without any effect on the overall chemical composition. Hence, the misorientation gradients and small-angle grain boundaries along the columnar grains are concluded to be determinant for the increased hardness. This microstructural improvement of mechanical properties is relatively simple to implement, thus offering a promising concept for novel coatings to be developed for potential use in friction and wear applications.

## Experimental Details

### Coating deposition

**Figure 9:**
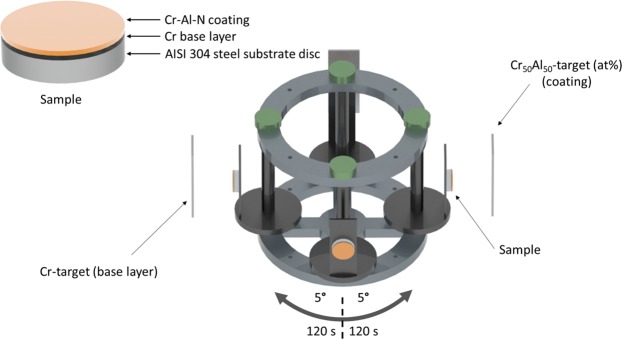
Schematic view of the HiPIMS-250 PVD chamber during deposition of multilayer-like Cr_1−x_Al_x_N single films.

AISI 304 steel discs with 30 mm diameter and 10 mm thickness were ground with SiC paper down to #2500 and polished with diamond suspensions of 6, 3 and 1 µm and colloidal silica for a mirror finish. After polished, the discs were cleaned in acetone ultrasonic bath for five minutes and blow dried. The discs were attached to a sample holder inside a HiPIMS-250 (Plasma-LIITS, Brazil) PVD chamber, 65 mm apart from the target surface. The sample holder was fixed to a carousel which, besides a planetary movement, could be programmed to oscillate in front of the targets with a desired amplitude and period (see Fig. 9)^26^. All substrates were let to oscillate with an amplitude of −5°/+5°, with the 0° position corresponding to the sample surface parallel to the target surface. The entire cycle from 0 to −5° to +5° and back to 0° took 120 s. Prior to deposition, the substrate surfaces were ion etched by Cr^+^ ions for 1 hour, using an Ar plasma at 400 °C and a substrate bias of −60 V.

After the ion etching step, a Cr base layer was deposited using a Cr target of 220 × 110 mm^2^ during 18 minutes with a working pressure of 2 mtorr, Ar flow of 40 sccm and temperature of 400 °C. The substrate DC bias was set to −60 V, which remained the same during the Cr_1−x_Al_x_N deposition for all samples. Last, under N_2_/Ar atmosphere (flow of 50 and 40 sccm, respectively, and 2 mtorr working pressure) the Cr_1−x_Al_x_N layers were deposited using a CrAl (50/50at%) target of 220 × 110 mm^2^ for 3 hours.

All the above etching and deposition steps used a True Plasma High pulse 4004 (TRUMPF Hüttinger, Germany) power supply operating at 900 W average power. The on-time was set to 200 µs for all experiments. Four different HiPIMS pulse frequencies were investigated: 200, 300, 400, and 500 Hz.

### Coating characterization

X-ray diffraction (XRD) was applied to phase analyses in the ϴ-2ϴ mode using a Rotaflex Ru200B (Rigaku, Japan) diffractometer equipped with rotative anode and monochromatized Cu Kα radiation (1.5418 Å). Crystallite size was determined using the Scherrer equation^27^, and Si standard powder sample was used to determine instrumental broadening.

The chemical composition of the Cr_1−x_Al_x_N films was determined by glow discharge optical emission spectroscopy (GDOES) depth profile analysis, which was performed using a Spectruma Analytik GmbH GDA 750 HR equipped with a 2.5 mm diameter anode and operating in DC excitation mode (constant voltage-constant current mode)^28^. Every sample was measured by triplicate. In each one, the glow was obtained in argon atmosphere (5.0 quality) and average discharge pressure of 5 × 10^−2^ hPa. The excitation parameters for the measurements were 1000 V and 12 mA and the sputtering rate was calculated so that the measuring depth was at least 75 µm. Quantitative profiles of Mass Concentration [%] vs Depth were obtained automatically using the standard WinGDOES software^28^.

Residual stress analyses were carried out using a MRD-XL (Panalytical, The Netherlands) diffractometer equipped with Mo-Kα radiation (0.7093 Å). The residual stresses were determined by the sin^2^ψ method using 7 ψ-tilts for each sample by varying sin^2^ψ from 0 to 0.9 with increments of 0.15^29^. The (422), (511) and (333) diffraction lines of fcc-Cr_1−x_Al_x_N were used for averaging the d_hkl,ψ_ − sin^2^ψ profiles in the stress analyses due to their high multiplicity and their benefits therefore with respect to the linearization of texture-induced scattering in the d_hkl,ψ_ − sin^2^ψ profiles.

The Cr_1−x_Al_x_N coating morphology and multilayer-like periodicities were investigated by Scanning Electronic Microscopy equipped with a Field Emission Gun (FEG-SEM). The measurements were conducted using a Field Emission Inspect F-50 (FEI, The Netherlands) electron microscope. The FEG-SEM images were acquired from the top surface and cross-section of the samples. Atomic Force Microscopy (AFM) was carried out using a Nanosurf Flex (Nanosurf, Switzerland) in order to measure the surface finishing after deposition in an area of 30 × 30 µm. Focused Ion Beam (FIB) was employed to prepare TEM lamellas. Transmission Electron Microscopy (TEM) images and crystallographic orientation maps by precession electron diffraction were carried out using a FEI TECNAI G2 LaB_6_ TEM microscope (FEI, The Netherlands) equipped with an ASTAR system. The orientation maps were processed using the MTEX Matlab tool. EDS mapping at the TEM was conducted using a JEM 2100 LaB_6_ (JEOL, USA) TEM microscope equipped with an Oxford EDS Detector (Oxford).

Cr_1−x_Al_x_N coating hardness were measured using instrumented nanoindentation tests at normal forces of 50 mN with a PB1000 mechanical tester (Nanovea, USA) equipped with a Berkovich diamond tip. The indenter was calibrated using a fused silica standard. The Oliver and Pharr equations were considered to calculate the hardness values^30^. At least 7 measurements were performed on top of each coating to determine an average value and its respective standard deviation.
